# Hybrid Endovascular Repair Combined With Surgical Hematoma Evacuation for a Giant Ruptured Spontaneous Femoral Artery Pseudoaneurysm

**DOI:** 10.7759/cureus.89687

**Published:** 2025-08-09

**Authors:** KaMan Ng, Nim Choi, Hongru Deng, Gangzhu Liang

**Affiliations:** 1 General Surgery, Conde S. Januário Hospital, Macao, CHN; 2 Vascular Surgery, Conde S. Januário Hospital, Macao, CHN

**Keywords:** covered stent, endovascular repair, hematoma evacuation, hybrid operation, spontaneous femoral artery pseudoaneurysm

## Abstract

Spontaneous femoral artery pseudoaneurysms (SFAPs) represent a rare vascular entity. We report the successful hybrid management of a large, wide-necked ruptured SFAP in an 85-year-old male. Computed tomography angiography (CTA) confirmed a massive pseudoaneurysm originating from the distal right superficial femoral artery (SFA) with severe circumferential arterial calcification. Given the prohibitive risks of conventional open repair due to advanced age, comorbidities, and lesion complexity, a hybrid approach was employed: endovascular repair with a self-expanding covered stent, followed by open surgical evacuation of the organized hematoma. This strategy achieved complete pseudoaneurysm repair, effective hematoma evacuation, and preserved distal perfusion without major complications. A brief review of the etiology, diagnostic considerations, and contemporary therapeutic algorithms is provided to contextualize this rare presentation.

## Introduction

Pseudoaneurysms of the femoral artery were historically most commonly associated with trauma occurring approximately 20 years prior [1]. However, with the increasing popularity of endovascular treatments, iatrogenic factors have emerged as the primary etiological cause [2]. These typically occur during vascular catheterization, vessel instrumentation, or due to the failure of percutaneous closure devices, with reported incidence rates of less than 1% [2,3]. Spontaneous femoral artery pseudoaneurysms (SFAPs) remain an exceedingly rare condition [4], whose pathogenesis appears distinct from traumatic or iatrogenic variants. The predominant etiology involves intrinsic vascular wall fragility or structural deterioration secondary to vasculitis, rather than direct vascular injury [5]. Treatment options for femoral artery pseudoaneurysms (FAPs) include open surgical repair, ultrasound-guided compression, ultrasound-guided thrombin injection, coil embolization, and covered stent placement [6,7]. However, management of SFAP requires a tailored, etiology-driven approach, as its underlying pathogenesis differs from that of traumatic or iatrogenic counterparts. Herein, we report a case of a massive ruptured SFAP successfully managed with a hybrid operation. This approach integrated endovascular covered stent implantation for definitive pseudoaneurysm exclusion and surgical decompression for comprehensive hematoma evacuation, achieving optimal anatomical and functional outcomes.

## Case presentation

**Figure 1:**
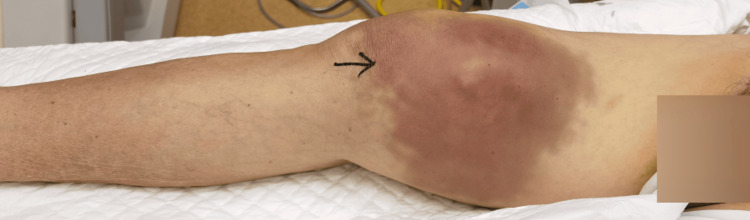
Severe tense swelling with subcutaneous ecchymoses was observed in the right distal thigh.

An 85-year-old male with a history of hypertension and dyslipidemia presented to the emergency department. He had no other significant comorbidities or a history of surgery. For three months, he had been experiencing insidious right thigh pain without trauma or vascular intervention, which acutely worsened over two days, accompanied by progressive distal right thigh swelling and increasing tenderness. On admission, the patient’s vital signs were stable, with a blood pressure of 130/85 mmHg and a heart rate of 90 beats per minute. He was afebrile. Physical examination revealed tense swelling with ecchymotic patches in the right distal thigh (Figure 1).

**Figure 2:**
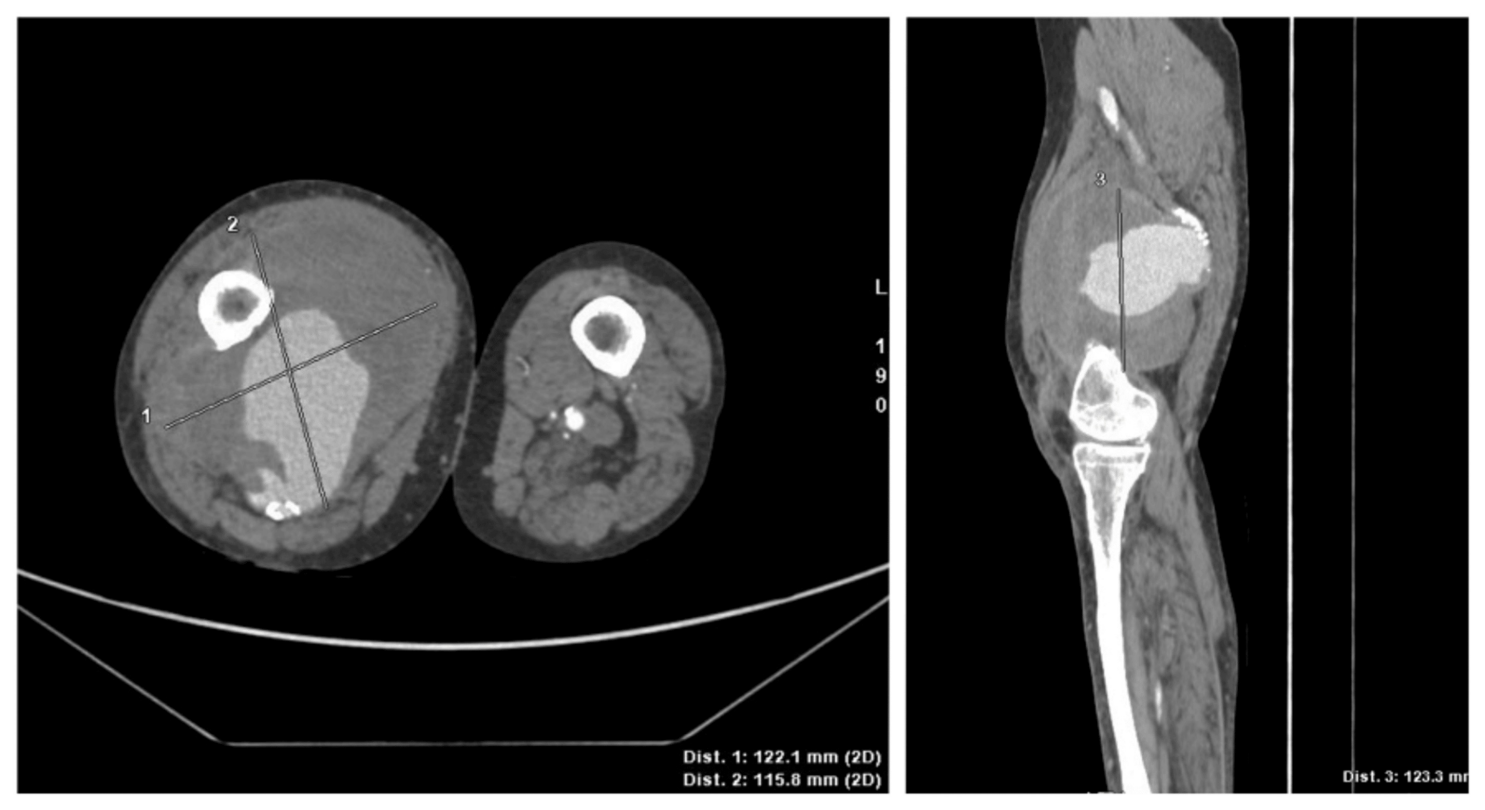
CTA demonstrated a large pseudoaneurysm from the right SFA, measuring 12.2 cm x 11.6 cm x 12.3 cm. Severe arterial calcification at the rupture site was demonstrated. CTA: computed tomography angiography; SFA: superficial femoral artery

The right popliteal and dorsalis pedis artery pulses were diminished but palpable, and the sensorimotor function of the right lower extremity was intact. Anemia persisted (hemoglobin: 5.3 g/dL), even though the patient had received a red blood cell transfusion. Duplex ultrasonography demonstrated a large pseudoaneurysm with mural thrombosis and turbulent flow. Computed tomography angiography (CTA) confirmed a 12.2 cm × 11.6 cm × 12.3 cm ruptured pseudoaneurysm of the right SFA, with severe arterial calcification at the rupture site (Figure 2).

**Figure 3:**
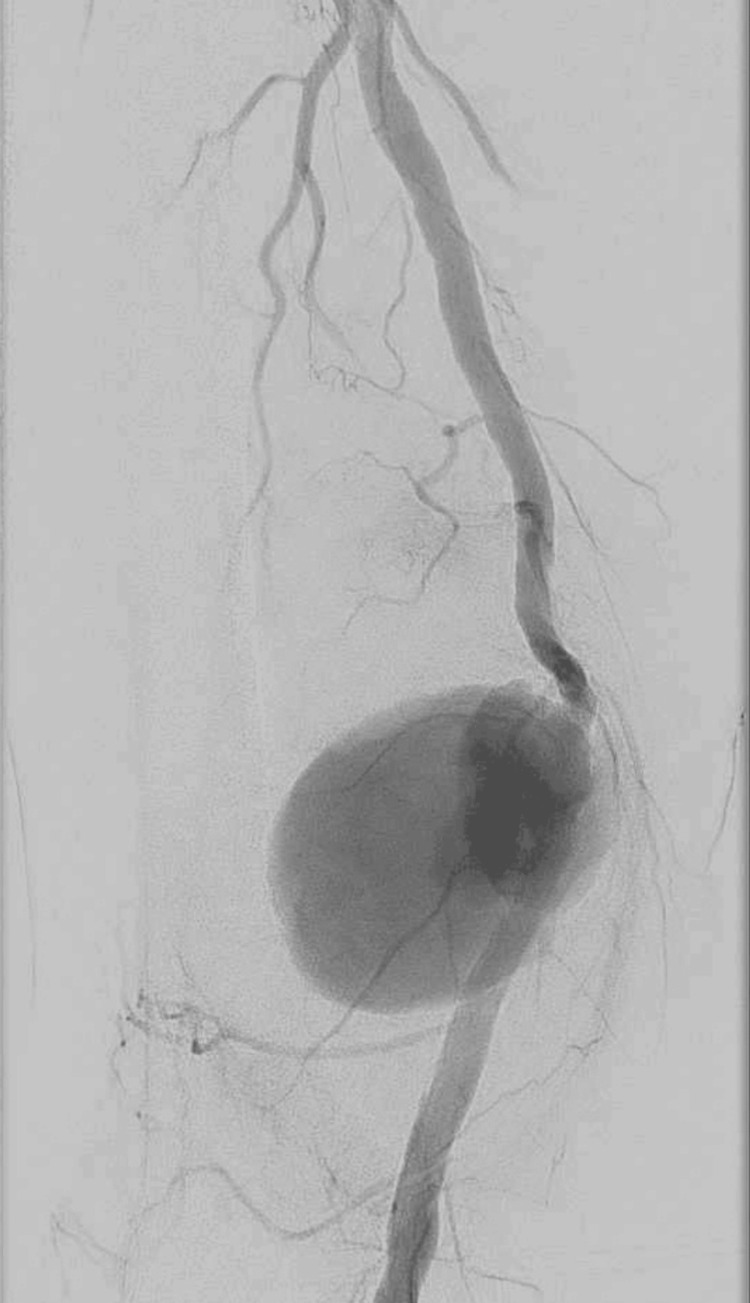
Angiography showed a large pseudoaneurysm in the distal segment of the right SFA. SFA: superficial femoral artery

Given the patient’s advanced age and complex arterial calcification, an emergent hybrid strategy was elected: endovascular covered stent repair of the arterial defect combined with surgical hematoma decompression. Antegrade access to the right common femoral artery was obtained. Angiography visualized a large distal SFAP (Figure 3).

**Figure 4:**
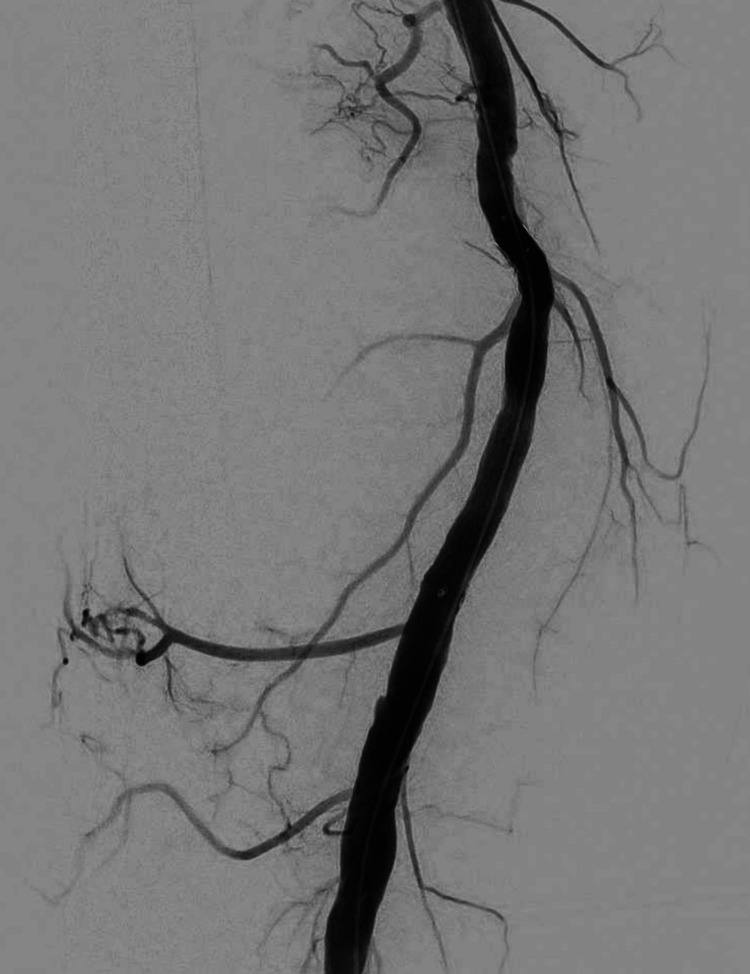
An 8×80 mm self-expanding covered stent was deployed in the right SFA, achieving complete occlusion of the pseudoaneurysm and maintaining distal runoff. SFA: superficial femoral artery

An 8-mm × 80-mm self-expanding covered stent (Fluency™ Plus, BD, NJ) was deployed, achieving complete pseudoaneurysm occlusion and preserving distal runoff (Figure 4).

**Figure 5:**
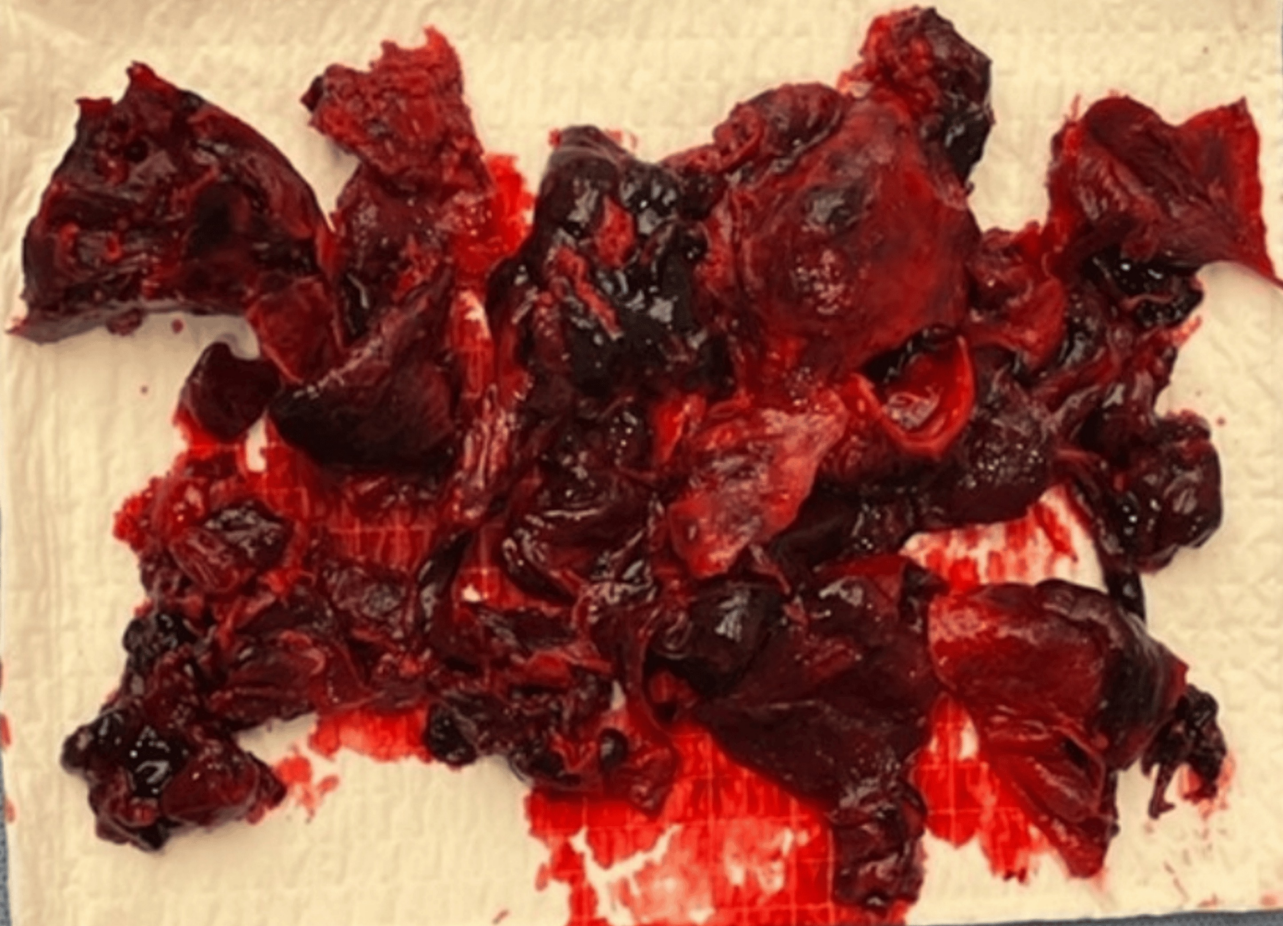
An amount of thrombotic material was surgically evacuated from the pseudoaneurysm sac.

Fasciotomy and thrombus evacuation (Figure 5) were performed to relieve compression of neurovascular structures and the stent graft.

**Figure 6:**
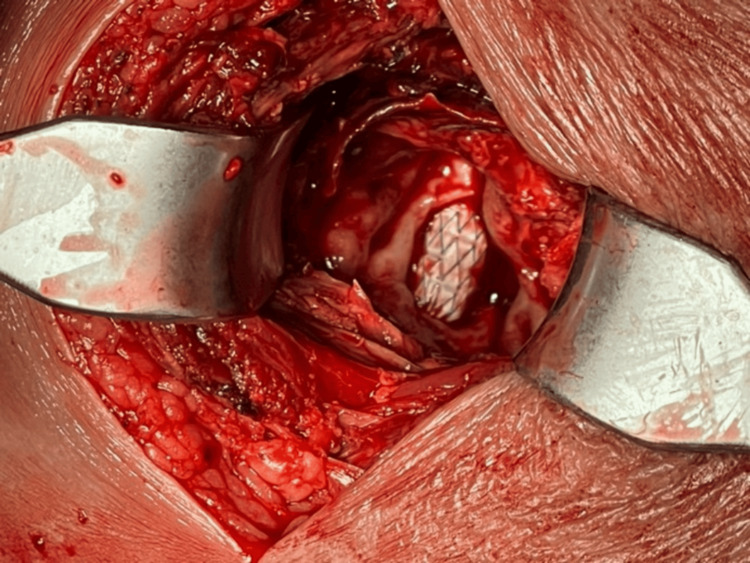
The distal segment of the right SFA was surgically exposed, revealing a 3-cm longitudinal laceration, and the covered stent was visualized. SFA: superficial femoral artery

Intraoperative inspection revealed a large arterial laceration with the covered stent visible through the defect (Figure 6).

The postoperative course was uncomplicated, and the patient was discharged on postoperative day 14. Long-term antiplatelet therapy with aspirin, 100 mg per day, was administered postoperatively. At two-year follow-up, he had no evidence of lower extremity ischemia or claudication, with patent flow through the SFA stent confirmed by imaging.

## Discussion

In this case report, we describe the successful management of a rare, large ruptured SFAP using a hybrid approach. The arterial defect was addressed with endovascular implantation of a self-expanding covered stent, while surgical hematoma evacuation was performed to prevent secondary compression of neurovascular structures.

Nowadays, FAPs typically arise as an iatrogenic complication following percutaneous endovascular procedures via femoral access, or less commonly from infectious, drug abuse, or traumatic etiologies [2,3]. SFAPs represent an extremely rare vascular pathology, as evidenced by isolated case reports in the literature [4,8]. In these reports, none of the SFAP cases were associated with direct trauma or prior intervention to the affected femoral artery. Pathogenetically, SFAPs may differ fundamentally from other FAPs. The underlying pathological substrate appears to be intrinsic arterial wall pathology-characterized by degenerative changes, structural weakness, or intrinsic vascular compromise-rather than extrinsic injury. Cardiovascular risk factors such as advanced age, hypertension, and diabetes promote maladaptive vascular remodeling through endothelial dysfunction and extracellular matrix degradation. These mechanisms collectively weaken the femoral arterial wall, predisposing it to spontaneous pseudoaneurysm formation. Congenital vascular anomalies have also been implicated as a rare cause, and in young patients, vasculitis or collagen disorders should be considered, highlighting the necessity of a genetic workup [4, 8-11]. This case report described an 85-year-old male with FAP in the absence of classic injury-induced risk factors. Clinical diagnosis confirmed an SFAP, with risk factors attributed to advanced age and underlying cardiovascular comorbidities (e.g., hypertension, dyslipidemia). These factors are presumed to compromise arterial wall integrity by inducing degenerative changes and structural fragility. Notably, significant calcification at the rupture site likely contributed to reduced wall elasticity, exacerbating vascular tearing by impairing the vessel's ability to withstand hemodynamic stress and promoting progressive pseudoaneurysm sac expansion.

Clinical manifestations of SFAPs often include a pulsatile mass, local swelling, pain, and, when ruptured, potential hemodynamic compromise. Multimodality imaging is typically integrated into the diagnostic workup: ultrasound for initial evaluation of aneurysm size, anatomical localization, and hemodynamic changes; CTA or magnetic resonance imaging to confirm SFAP dimensions, assess relationships with surrounding tissues, evaluate vascular wall condition, and exclude concomitant aneurysms; and angiography for detailed vascular mapping, distal runoff assessment, and simultaneous endovascular intervention. In this case, the patient presented with insidious right thigh pain for three months, without a history of trauma or vascular intervention. Acute-onset distal right thigh swelling, pain, and ecchymotic lesions developed over two days, accompanied by a persistent decline in hemoglobin, which suggested rupture of a pseudoaneurysm. Duplex ultrasonography and CTA demonstrated a giant femoral pseudoaneurysm with rupture, confirming the diagnosis.

Management of SFAPs requires individualized strategies balancing invasiveness and durability. Formal clinical guidelines for FAPs/SFAPs remain unavailable. Pseudoaneurysm management strategies range from conservative measures to invasive interventions, including open surgical repair (direct suture, patch angioplasty, or bypass grafting), endovascular techniques (coil embolization, stent-graft repair), and ultrasound-guided therapies (compression or thrombin injection) [6,7,12]. Although open repair remains the gold standard for definitive treatment, endovascular approaches have gained increasing preference in high-risk patient populations due to their minimally invasive nature and favorable perioperative outcomes. The distinct pathogenesis of SFAPs versus iatrogenic or trauma-induced FAPs mandates individualized management. Thus, treatment optimization relies on case-specific factors-age, comorbidities, pseudoaneurysm characteristics, and etiology. For this elderly patient with complex comorbidities and severe arterial calcification, a hybrid surgical approach was deemed the most suitable strategy. Open surgical options (direct patch repair or vascular bypass) were considered infeasible due to technical challenges from severe mural calcification and the risk of compromised anastomotic integrity. Intraoperative findings revealed severe atherosclerosis and calcification of the SFA. Endovascular covered stent repair of the pseudoaneurysm was preferred, as it offered a minimally invasive approach with lower perioperative morbidity while effectively sealing the rupture site. Selection of the appropriate covered stent warrants particular consideration in the SFA and popliteal segments. The human femoral-popliteal arteries possess unique structural and mechanical properties to function in the dynamic biomechanical environment of the lower extremity, with the SFA and popliteal artery undergoing significant conformational changes during movement and being exposed to multiple dynamic stresses such as compression, elongation, torsion, and bending [13]. These mechanical demands necessitate the use of a self-expanding covered stent with superior flexibility and radial strength to maintain long-term patency and structural integrity. Balloon-expandable covered stents are generally contraindicated in this anatomical region due to the complex biomechanical environment. The covered stent selected in our case was the Fluency® Plus (BD, NJ) covered stent, the only self-expanding covered stent available at our center. This flexible covered stent consists of a nitinol stent framework encapsulated in expanded polytetrafluoroethylene (ePTFE), combining mechanical durability with biocompatible sealing properties. Surgical hematoma evacuation was also deemed essential, as the massive pseudoaneurysm posed risks of neurovascular compression and potential infectious complications.

Despite therapeutic interventions, recurrence has been documented [4], highlighting the critical need for rigorous postoperative follow-up. The patient has been followed in the vascular surgery outpatient clinic for two years without complications such as stent occlusion, stent fracture, hemorrhage, infection, or other procedure-related events. This outcome validates the rationale of our treatment strategy and demonstrates the favorable flexibility and long-term patency of the selected covered stent.

## Conclusions

SFAPs are rare entities characterized by intrinsic arterial wall fragility, which distinguishes them from traumatic or iatrogenic pseudoaneurysms. The hybrid approach combined endovascular repair with surgical hematoma evacuation, addressing the patient’s complex arterial calcification and high-risk profile. The treatment effectively repaired the pseudoaneurysm, preserved distal perfusion, and prevented potential complications. This case highlights the importance of individualized management strategies for SFAP, emphasizing the need for tailored approaches based on patient-specific factors and the unique pathogenesis of this rare condition.
